# The Use of Session RPE to Monitor the Intensity of Weight Training in Older Women: Acute Responses to Eccentric, Concentric, and Dynamic Exercises

**DOI:** 10.1155/2014/749317

**Published:** 2014-04-13

**Authors:** Sandro S. Ferreira, Kleverton Krinski, Ragami C. Alves, Mariana L. Benites, Paulo E. Redkva, Hassan M. Elsangedy, Cosme F. Buzzachera, Tácito P. Souza-Junior, Sergio G. da Silva

**Affiliations:** ^1^Department of Physical Education, Federal University of Parana, Caixa Postal 92, JD Botânico, 80215-370 Curitiba, PR, Brazil; ^2^Federal University of Sao Francisco Valley, 56304205 Petrolina, PE, Brazil; ^3^Center for Health Sciences, Federal University of Rio Grande do Norte, Caixa Postal 3000, Lagoa Nova, 59078970 Natal, RN, Brazil; ^4^University Norte of Parana, Caixa Postal 675, JD Piza, 86041-100 Londrina, PR, Brazil

## Abstract

The rating of perceived exertion (RPE) is ability to detect and interpret organic sensations while performing exercises. This method has been used to measure the level of effort that is felt during weight-training at a given intensity. The purpose of this investigation was to compare session RPE values with those of traditional RPE measurements for different weight-training muscle actions, performed together or separately. Fourteen women with no former weight-training experience were recruited for the investigation. All participants completed five sessions of exercise: familiarization, maximum force, concentric-only (CONC-only), eccentric-only (ECC-only), and dynamic (DYN = CONC + ECC). The traditional RPE method was measured after each series of exercises, and the session RPE was measured 30 min after the end of the training session. The statistical analyses used were the paired *t*-test, one-way analysis of variance, and repeated measures analysis of variance. Significant differences between traditional RPE and session RPE for DYN, CONC, and ECC exercises were not found. This investigation demonstrated that session RPE is similar to traditional RPE in terms of weight-training involving concentric, eccentric, or dynamic muscle exercises, and that it can be used to prescribe and monitor weight-training sessions in older subjects.

## 1. Introduction

Weight training has been commonly recommended for elderly subjects [1, 2]. Based on this premise, it is important to consider that weight training is conducted by eccentric, concentric, and isometric muscle actions, which can be performed together or separately [3, 4]. Thus, an action involving the generation of tension in the muscle, without external changes in the muscle length or joint angle with movement, is characterized as isometric action (ISOM). On the other hand, muscular tension related to decreases in the muscle length and joint angle is called concentric action (CONC), and when the generation of force causes stretching of the muscle length and angle joint, it is known as eccentric action (ECC) [5]. Most weight training programs primarily include dynamic exercises with both CON and ECC muscle actions, whereas ISOM actions play a secondary role [3].

Over the past decades, several investigations have demonstrated that a routine of weight training involving ECC and CONC muscle actions, in a combined or isolated manner, contributes to improved cardiovascular health, muscle strength, and functional capacity in the elderly [6, 7]. However, the benefits derived from these muscle actions are dependent on correct application and intensity control, including the numbers of sets and repetitions, rest intervals between sets, resistance used (load), and execution of velocity [3, 4]. Thus, adjustment and manipulation of each of these variables provide intensity capable of inducing a muscle response according to individual needs [8]. In this sense, developing strategies to measure the intensity utilized for weight training represents a considerable challenge.

Previous studies have used the rating of perceived exertion (RPE) as a method to measure the level of effort that is felt during weight training at a given intensity [9, 10]. The rating of perceived exertion (RPE) can be defined as the ability to detect and interpret organic sensations while performing exercises [11]. In fact, the recent guidelines proposed by the American College of Sports Medicine for physical activity and public health in older adults have suggested scales of perceived exertion of 0–10 for measuring exertion during weight training [12]. Thus, the traditional method of measuring RPE is commonly used immediately after the execution of each exercise, reflecting various measurement points according to the amount of exercise performed. To facilitate the measurement and quantification of exercise intensity, Foster et al. [13] developed the method of subjective perception of exertion during a training session (session RPE), used to monitor various exercise types, particularly weight training. This method is based on a simple question that the subject responds to 30 min after the end of the training session: “What level of exertion did you feel in your body during the training session?” This reflects the feeling of global exertion experienced during the entire session.

A number of studies [9, 14, 15] have shown that a single session RPE may accurately reflect the intensity of a weight training session. In addition, this method allows an easy and reliable resistance training program manipulation required for continual increases in strength, including all workout stages in a single measure for the individual after each exercise session [9, 14]. In addition, it allows greater training synchronicity between a training regimen prescribed by a trainer and the actual intensity at which the subjects train. However, no study has investigated the application of session RPE in older individuals for different muscle actions used in weight training. Consequently, a better understanding of the session RPE in older individuals can provide valuable insight to health care professionals for prescribing weight training comprising different muscle actions. Therefore, the aim of this study was to compare values between session RPE and traditional measurement of RPE for different muscle actions performed together or separately in weight training.

## 2. Methodology

### 2.1. Subjects

A convenience sample of 14 older women met the inclusion criteria and gave their written consent to participate in this study. All were classified as either physically active (regular exercise ≥ 3 days·week^−1^) or as having no former weight training experience. The inclusion criteria were (a) between the ages of 60 and 75 years; (b) ability to take part in regular physical exercise; (c) negative responses to all questions in the Physical Activity Readiness Questionnaire (PAR-Q); (d) a body mass index (BMI) between 18.5 and 30 kg·m^−2^; and (e) a personal statement of not having smoked in the last 12 months. Criteria for exclusion included the presence of cardiovascular, metabolic, or orthopedic disease or any other contraindications as determined by a medical history from the previous 12 months. The study was approved by the Research Ethics Committee of the Department of Health Sciences at the Federal University of Paraná (UFPR) in Curitiba, Brazil.

### 2.2. Experimental Design

The experimental design of this study can be classified ascross-sectional [16]. All subjects completed five sessions of exercise: (a) sample screening and familiarization, (b) determination of 1RM (Repetition Maximum), and (c) three sessions of weight training conducted on different days, with 24–48 h between sessions, with the orders counterbalanced. Each session involved a different protocol: (1) ECC-only exercise, which consisted only of stretching of the muscle length and angle joint; (2) CONC-only exercise, which consisted of only the shortening phase of the muscle and angle joint; (3) DYN = CONC + ECC, which consisted of both a lengthening and shortening phase. The RPE and session RPE were recorded during each experimental session. Thus, the independent variable was muscle action (ECC-only, CONC-only, and DYN), whereas the dependent variables were RPE in-task and session RPE to the task. Subjects were advised not to consume alcohol, caffeine, or practice vigorous physical activity 24 h prior to each test.

### 2.3. Familiarization Session

To facilitate the elderly's understanding of the experimental procedures, subjects performed a familiarization session. Instructions were provided on the correct execution and proper form of the prescribed exercises, mainly regarding the appropriate posture, utilization of constant range of motion, and movement speed. In addition, information concerning the use of the OMNI Resistance Exercise Scale (OMNI-RES), which ranges from 0 to 10, was provided to the subjects [17] with the following specific instructions: “Please use this scale to translate into numbers your perception of exertion while exercising. The numbers on the scale represent a range of feelings from extremely easy 0 (low anchor) to extremely hard 10 (higher anchor). To help you select a number that corresponds to your perceptions regarding the exercise, consider the following: when the exercise seems extremely easy respond with a 0; on the other hand, when the exercise seems extremely hard, respond with a 10.” Thus, subjects were instructed to use their memory of the least and greatest effort experienced while lifting weights to establish a visual-cognitive link with low and high perceptual anchors of the OMNI-RES, respectively. Furthermore, each subject was asked to select a load (light, moderate, or high) based on perceived exertion ratings on the OMNI-RES scale (0–10) with respect to all of the exercises (chest press, leg extension, lat pulldown, leg curl, and lateral shoulder raise).

### 2.4. Determination of 1RM

The 1RM testing session began with a specific warm-up, consisting of five to eight repetitions of each exercise using a self-selected, light weight. After this initial procedure, subjects were requested to take 5 min for passive recovery. Determination of the 1RM load was executed over a maximum of four attempts for each exercise, with 3 min of rest. Therefore, in each attempt, weight was successively added until only one repetition (completed with good form) was successfully lifted or until the participant indicated that he could not lift any more weight. Thus, the 1RM test was performed with each resistance exercise (chest press, leg extension, lat pulldown, leg curl, and lateral shoulder raise). To reduce the possible cumulative effect of fatigue on 1RM performance, the exercise order was alternated between upper and lower body exercises to allow greater recovery. All subjects were familiarized with the experimental procedures and were subjected to different exercise intensities (low, moderate, and high) for all the previously mentioned exercises. Consequently, this facilitated estimation of the initial loads and subsequent increments on 1RM tests.

### 2.5. Weight Training Sessions

All subjects completed three sessions of weight training, which were conducted on different days and with the orders counterbalanced. Thus, each weight training session was categorized according to the type of muscle action (CONC-only, ECC-only, and DYN). To guarantee the correct execution of the exercise and load application, each weight training session and each exercise were supervised by two experienced fitness instructors. In addition, during the CONC-only and ECC-only weight training sessions, subjects received help from two instructors, which allowed for these exercises to be performed in an isolated manner. Therefore, during the CONC-only session, subjects lifted the weight for complete execution of each movement (shortening of muscle length and angle joint), whereas two fitness instructors reduced the weight during the eccentric phase with the help of hands. On the other hand, during the ECC-only session, two fitness instructors lifted the weight for each repetition, and then the subject used strength to lower the weight (stretching of muscle length and angle joint). During the DYN session, both muscle actions (ECC and CONC) were performed without the help of instructors, only supervision to verify the correct execution of the exercise.

The weight training sessions (CONC-only, ECC-only, and DYN) consisted of uniarticular and multiarticular exercises, free weights and machines (Nakagym, São Paulo, Brazil), for large and small muscle groups, based on three sets of 8 to 10 repetitions each. Thus, all weight training sessions adopted the following exercises: chest press, leg extension, lat pulldown, leg curl, and lateral shoulder raise, with the order alternating between upper and lower body exercises. All exercises were performed on machines with the exception of the lateral shoulder raise, which used free weights. The intensity of each weight training session was classified as follows: CONC-only (70% 1RM); ECC-only (90% 1RM); and DYN (70% 1RM). According to Hortobagyi and Katch [18], 90% 1RM with the eccentric action is equivalent to 70% 1RM with the concentric force. The execution speed of the exercises was controlled by verbal commands from the evaluator, such that the subject maintained a concentric to eccentric phase ratio of 2 : 2 s, in accordance with the procedures suggested by Kraemer and Ratamess [3].

### 2.6. Rating Perception of Exertion

The RPE was used after each series of exercises (traditional measurement). Session RPE was measured 30 min after the end of the training session following procedures described by Foster et al. [13]. The subject was presented with the OMNI-RES scale and asked to answer the following question: “What level of exertion did you feel in your body during the training session?” Subjects were instructed to consider only the overall perception of exertion. While the subjects were waiting, they were allowed to drink water as they wished, but they were not allowed to perform other tasks, such as eating or showering.

### 2.7. Statistical Analysis

To characterize those taking part in the study, descriptive statistics using means ± standard deviations (SD) were performed on the data collected. A paired *t*-test was used to measure differences between the traditional RPE and session RPE means. To compare perceptual responses between training sessions for the mean RPE and session RPE, one-way analysis of variance (ANOVA) was used. To analyze perceptual responses during the exercise session, repeated measures ANOVA was used. Because violations in the assumption of sphericity appeared, such were addressed using Greenhouse-Geisser corrections. The significance level adopted for the analysis was *P* < 0.05. Data were analyzed statistically using SPSS software (version 17.0).

## 3. Results

The corresponding age, anthropometric measurement, and 1RM percentage values for DYN, CONC, and ECC are shown in Table 1 as means ± standard deviations (SD).

**Figure 1:**
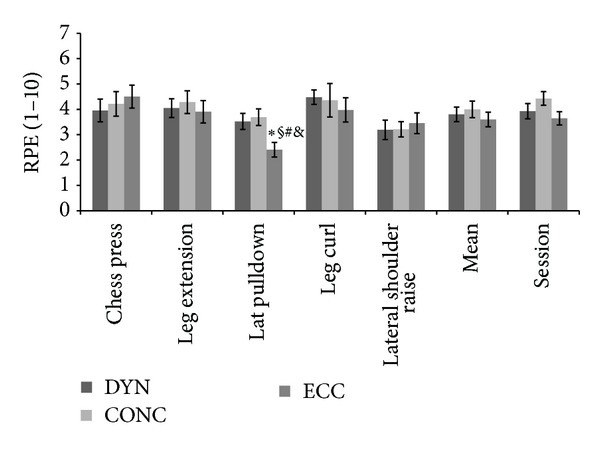
Significant differences: (*) Chess Press × Lat pulldown; (§) Leg Extension × Lat pulldown; (#) Mean × Lat pulldown; (&) Session × Lat pulldown.

Figure 1 shows the perceptual responses of the muscle exercises expressed as means ± SD. Significant differences between traditional RPE and session RPE for DYN (*P* = 0.626), CONC (*P* = 0.084), and ECC (*P* = 0.983) were not found. Significant differences were also not detected between mean RPE (*F*_(2,26)_ = 0.412, *P* = 0.667, *n*_*p*_^2^ = 0.031) and session RPE (*F*_(3.619,26)_ = 1.698, *P* = 0.203, *n*_*p*_^2^ = 0.116) for the training sessions analyzed. For the perceptual responses during the session, differences were only observed in eccentric action (*F*_(3.389,26)_ = 4.921, *P* = 0.004, *n*_*p*_^2^ = 0.275).

## 4. Discussion

The benefits of weight training on neuromuscular aging have been widely presented in the literature, in theoretical and practical studies [19, 20]. However, in practice, few people adhere to a regular training program in order to obtain these benefits [21]. The high intensity of weight training programs during the early stages is one of the factors hindering the subjects' adherence to regular exercise [21]. This fact may contribute to high RPE and influence the decision of subjects to keep up regular exercise [22].

Session RPE is a modification of the traditional RPE scale. The difficulty of using conventional methods, such as measurements of heart rate, percentage of maximum oxygen consumption, and lactate threshold during weight training, has motivated researchers to use session RPE to facilitate and quantify the measurement of perceptual responses to exercise using weights [15]. The study by Day et al. [9] measured the session RPE during low, moderate, and high intensity weight training and the required reliability of the method in adult subjects. With the same perspective, other studies used the session RPE for exercising with weights.

In overweight and obese children, McGuigan et al. [23] identified no significant difference between the session RPE and traditional RPE. Sweet et al. [15] found consistent results between the session RPE used for aerobic exercise and that used for weight training. Pritchett et al. [24] studied high resistance (90% RM) and moderate (60% RM) exercise intensity in adult men and found differences between the mean RPE and session RPE for low intensity exercise. However, the authors suggested that the protocol used (multiple sets until exhaustion) may have been primarily responsible for the difference.

The investigations performed so far consider the session RPE to be a useful and important tool for exercise with weights [15, 25]. However, no studies in older populations or on different muscle actions were performed. Therefore, the aim of this study was to compare session RPE and traditional RPE measurement values for different muscle actions performed together or separately for weight training conducted in elderly women.

The results of this study detected no differences between traditional RPE and session RPE for DYN (*P* = 0.626), CONC (*P* = 0.084), and EXC (*P* = 0.983) sessions, corroborating data from previous research using dynamic actions [9, 25]. These findings agree with previous investigations, which showed similarity between traditional RPE and session RPE.

After analyzing the results of the traditional RPE and comparing them with the mean final and session RPE, differences were found only for the lat pulldown exercise session. It is speculated that the higher absolute overload for eccentric action (90% 1RM) and the characteristics of the lat pulldown exercise used to assign overhead, compared with other exercises that sustain overhead, were the factors driving this significant difference. However, no studies have observed similar responses.

The prescription of each CONC, EXC, and DIN exercise session was conducted according to the American College of Sports Medicine's model of weight training progression for elderly subjects [2, 26, 27]. Although the overhead used is equivalent to 70% of the maximum strength, the perceptual responses obtained were approximately 4 on the OMNI scale, which represents a somewhat easy perceived exertion. According to the authors [28, 29], during weight training, the percentage of overhead used in the exercises (% 1RM) did not directly reflect the effort of the subjects in the exercises, because variables such as number of repetitions, execution time of the exercises, and rest intervals between sets influenced the interpretation of this effort.

No differences in RPE (mean final or session) were observed among the different muscle action responses. This result should be considered when prescribing training for elderly subjects, because the eccentric activity can promote increased strength and muscle mass with less of a response in heart rate, systolic blood pressure, diastolic blood pressure, and double product, compared with concentric and dynamic exercise [30–33].

## 5. Conclusion

This research showed that perceptual responses observed using the session RPE method were similar to those using the traditional RPE method in weight training involving concentric, eccentric, and dynamic muscle actions. This result enables the use of a method with easy application and low financial cost for monitoring the intensity of exercises in older subjects.

In weight training for elderly beginners, the session RPE can assist in prescribing exercise to avoid high intensities that promote cardiovascular overload and muscle damage and dispel the subject to regular exercise and low intensities do not provide inadequate physiological stimuli to the proposed objectives. However, a limitation of this study was the lack of an additional training session, involving the same variables, to test the reliability of the method in elderly subjects.

## Figures and Tables

**Table 1:** Anthropometric variables, 1RM tests, and training loads.

Variables	Average ± SD	Variables	1RM	70%	90%
Age (year)	68.5 ± 4.6	Chess press (kg)	22.23 ± 6.2	16.7 ± 5.6	21.1 ± 7.2
Weight (kg)	63.0 ± 12.0	Leg extension (kg)	59.62 ± 14.7	40.8 ± 9.5	51.5 ± 12.2
Height (cm)	154 ± 0.06	Lat pulldown (kg)	35.85 ± 6.6	25.4 ± 4.5	32.3 ± 5.8
BMI (kg·m^−2^)	26.1 ± 3.5	Leg curl (kg)	23.31 ± 9.6	17.2 ± 6.7	20.8 ± 7.5
		Lateral shoulder raise (kg)	4.08 ± 0.7	2.7 ± 0.6	3.2 ± 0.7

BMI: body mass index; data are expressed as means ± SD.
